# Supplementary material for: Obstetric admission and maternal mortality in the intensive care unit in Africa: A systematic review and meta-analysis
Source: PLoS One. 2025 Apr 16;20(4):e0320254. doi: 10.1371/journal.pone.0320254 (PMC12002433; doi:10.1371/journal.pone.0320254)
Supplement: S1 File — (DOCX) [file pone.0320254.s001.docx]

**Critical Appraisal**

Table: Critical appraisal checklist of quantitative studies of obstetric admission in the ICU in Africa. (1 =yes, 0=no/not mentioned); total score=9

| Studies | Q1 | Q2 | Q3 | Q4 | Q5 | Q6 | Q7 | Q8 | Q9 | Total score | Percent (%) |
| --- | --- | --- | --- | --- | --- | --- | --- | --- | --- | --- | --- |
| Author A, et al (1) | Y | Y | Y | Y | Y | Y | N | Y | Y | 8/9 | High |
| Author B, et al (2) | Y | Y | N | Y | Y | Y | N | Y | Y | 7/9 | Moderate |
| Author C, et al (3) | Y | Y | Y | Y | Y | Y | N | Y | Y | 7/8 | Moderate |
| Author D, et al (4) | Y | Y | N | Y | Y | Y | N | Y | Y | 7/9 | Moderate |
| Author E, et al (5) | Y | Y | N | Y | Y | Y | N | Y | Y | 7/9 | Moderate |
| Author F., et al (6) | Y | Y | N | Y | Y | Y | N | Y | Y | 7/9 | Moderate |
| Author G., et al (7) | Y | Y | N | Y | Y | Y | N | Y | Y | 7/9 | Moderate |
| Author H (8) | Y | Y | Y | Y | Y | Y | N | Y | Y | 8/9 | High |
| Author I, et al (9) | Y | Y | N | Y | Y | Y | N | Y | Y | 7/9 | Moderate |
| Author J., et al (10) | Y | Y | N | Y | Y | Y | N | Y | Y | 7/9 | Moderate |
| Author K., et al (11) | Y | Y | N | Y | Y | Y | N | Y | Y | 7/9 | Moderate |

1 =yes, 0=no/not mentioned, NA= not applicable; total score=9

Notes:

Q1 - Was the sample frame appropriate to address the target population?

Q2 - Were study participants sampled in an appropriate way?

Q3 - Was the sample size adequate?

Q4 - Were the study subjects and the setting described in detail?

Q5 - Was the data analysis conducted with sufficient coverage of the identified sample?

Q6 - Were valid methods used for the identification of the condition?

Q7 - Was the condition measured in a standard, reliable way for all participants?

Q8 - Was there appropriate statistical analysis?

Q9 - Was the response rate adequate, and if not, was the low response rate managed appropriately?

Abbreviations: Y, yes; N, no; U, unclear

**References**

1. Anane-Fenin B, Agbeno EK, Osarfo J, Anning DAO, Boateng AS, Ken-Amoah S, et al. A ten-year review of indications and outcomes of obstetric admissions to an intensive care unit in a low-resource country. PLOS ONE. 2021 Dec 31;16(12):e0261974.

2. Asudo DrFD, Akitoye OA, Abdullahi HI. Obstetric Patients Requiring Intensive Care: Prevalence, Clinical Characteristics and Outcome in a Tertiary Care Institute in Nigeria. EAS J Anesthesiol Crit Care. 2022 Aug 19;4(4):52–63.

3. Embu HY, Isamade ES, Nuhu SI, Oyebode TA, Kahansim ML. Obstetric admissions in a general intensive care unit in north-central Nigeria. Tropical Journal of Obstetrics and Gynaecology. 2016;33(1):14–20.

4. Imarengiaye CO, Isesele TO. Intensive care management and outcome of women with hypertensive diseases of pregnancy. Nigerian Medical Journal. 2015 Oct;56(5):333.

5. John CO, Alegbeleye JO, Oppah IC. A Five-year Review of the Pattern and Outcome of Obstetric Admissions into the Intensive care unit of a University Teaching Hospital in Southern Nigeria. International Journal of Science and Research Archive. 2022;5(2):155–62.

6. Ntuli TS, Ogunbanjo G, Nesengani S, Maboya E, Gibango M. Obstetric intensive care admissions at a tertiary hospital in Limpopo Province, South Africa. Southern African Journal of Critical Care. 2015;31(1):8–10.

7. Mideksa T, Mekonnen T, Mengiste B. Outcomes and Associated Factors of Mothers Admitted to Intensive Care Unit During Pregnancy and Postpartum at Saint Paul’s Hospital Millennium Medical College, Addis Ababa, Ethiopia. 2022.

8. Motiang M. Obstetric patients admitted to the intensive care unit of Dr George Mukhari Academic Hospital, Ga-Rankuwa, South Africa. Southern African Journal of Critical Care. 2017 Aug 2;33(1):12–4.

9. Prin M, Kadyaudzu C, Aagaard K, Charles A. Obstetric admissions and outcomes in an intensive care unit in Malawi. International Journal of Obstetric Anesthesia. 2019 Aug 1;39:99–104.

10. Rudakemwa A, Cassidy AL, Twagirumugabe T. High mortality rate of obstetric critically ill women in Rwanda and its predictability. BMC Pregnancy Childbirth. 2021 May 25;21(1):401.

11. Igbaruma S, Olagbuji B, Aderoba A, Kubeyinje W, Ande B, Imarengiaye C. Severe maternal morbidity in a general intensive care unit in Nigeria: clinical profiles and outcomes. International Journal of Obstetric Anesthesia. 2016 Dec 1;28:39–44.
