# Supplementary material for: Obstetric admission and maternal mortality in the intensive care unit in Africa: A systematic review and meta-analysis
Source: PLoS One. 2025 Apr 16;20(4):e0320254. doi: 10.1371/journal.pone.0320254 (PMC12002433; doi:10.1371/journal.pone.0320254)

- **Pubmed= 38 results, with search strategy:**

**((Obstetrics OR obstetric OR pregnancy complication OR postpartum complication OR maternal complication OR maternal morbidity OR near miss OR ("Obstetric Labor Complications/diagnosis"[Mesh] OR "Obstetric Labor Complications/epidemiology"[Mesh] OR "Obstetric Labor Complications/mortality"[Mesh])) AND (ICU OR Intensive care unit OR critical care unit OR ("Intensive Care Units/statistics and numerical data"[Mesh] OR "Intensive Care Units/trends"[Mesh]))) AND (Africa)** Filters: **Full text, Observational Study, English, from 2009/1/1 - 2023/11/30**

(("obstetric"[All Fields] OR "obstetrically"[All Fields] OR "obstetrics"[MeSH Terms] OR "obstetrics"[All Fields] OR "obstetrical"[All Fields] OR ("obstetric"[All Fields] OR "obstetrically"[All Fields] OR "obstetrics"[MeSH Terms] OR "obstetrics"[All Fields] OR "obstetrical"[All Fields]) OR ("pregnancy complications"[MeSH Terms] OR ("pregnancy"[All Fields] AND "complications"[All Fields]) OR "pregnancy complications"[All Fields] OR ("pregnancy"[All Fields] AND "complication"[All Fields]) OR "pregnancy complication"[All Fields]) OR (("postpartum period"[MeSH Terms] OR ("postpartum"[All Fields] AND "period"[All Fields]) OR "postpartum period"[All Fields] OR "postpartum"[All Fields]) AND ("complicances"[All Fields] OR "complicate"[All Fields] OR "complicated"[All Fields] OR "complicates"[All Fields] OR "complicating"[All Fields] OR "complication"[All Fields] OR "complication s"[All Fields] OR "complications"[MeSH Subheading] OR "complications"[All Fields])) OR (("maternally"[All Fields] OR "maternities"[All Fields] OR "maternity"[All Fields] OR "mothers"[MeSH Terms] OR "mothers"[All Fields] OR "maternal"[All Fields]) AND ("complicances"[All Fields] OR "complicate"[All Fields] OR "complicated"[All Fields] OR "complicates"[All Fields] OR "complicating"[All Fields] OR "complication"[All Fields] OR "complication s"[All Fields] OR "complications"[MeSH Subheading] OR "complications"[All Fields])) OR (("maternally"[All Fields] OR "maternities"[All Fields] OR "maternity"[All Fields] OR "mothers"[MeSH Terms] OR "mothers"[All Fields] OR "maternal"[All Fields]) AND ("epidemiology"[MeSH Subheading] OR "epidemiology"[All Fields] OR "morbidity"[All Fields] OR "morbidity"[MeSH Terms] OR "morbid"[All Fields] OR "morbidities"[All Fields] OR "morbids"[All Fields])) OR ("near"[All Fields] AND "miss"[All Fields]) OR ("obstetric labor complications/diagnosis"[MeSH Terms] OR "obstetric labor complications/epidemiology"[MeSH Terms] OR "obstetric labor complications/mortality"[MeSH Terms])) AND ("intensive care units"[MeSH Terms] OR ("intensive"[All Fields] AND "care"[All Fields] AND "units"[All Fields]) OR "intensive care units"[All Fields] OR "icu"[All Fields] OR ("intensive care units"[MeSH Terms] OR ("intensive"[All Fields] AND "care"[All Fields] AND "units"[All Fields]) OR "intensive care units"[All Fields] OR ("intensive"[All Fields] AND "care"[All Fields] AND "unit"[All Fields]) OR "intensive care unit"[All Fields]) OR ("intensive care units"[MeSH Terms] OR ("intensive"[All Fields] AND "care"[All Fields] AND "units"[All Fields]) OR "intensive care units"[All Fields] OR ("critical"[All Fields] AND "care"[All Fields] AND "unit"[All Fields]) OR "critical care unit"[All Fields]) OR ("intensive care units/statistics and numerical data"[MeSH Terms] OR "intensive care units/trends"[MeSH Terms])) AND ("africa"[MeSH Terms] OR "africa"[All Fields] OR "africa s"[All Fields] OR "africas"[All Fields]))

- **Web of Science= 120 results**


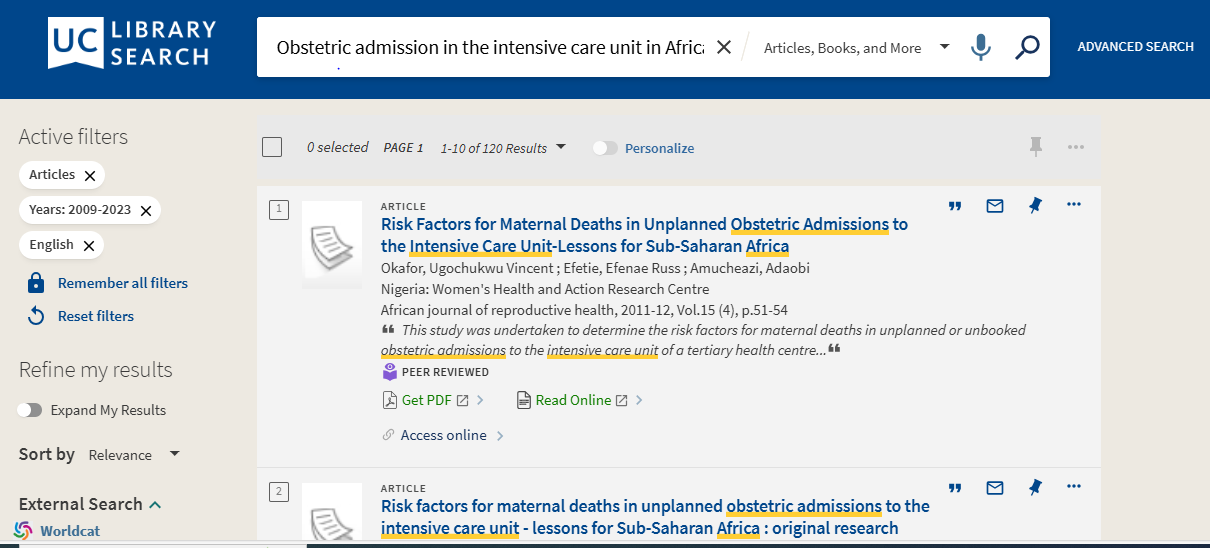


- **Hinary= 185 results**


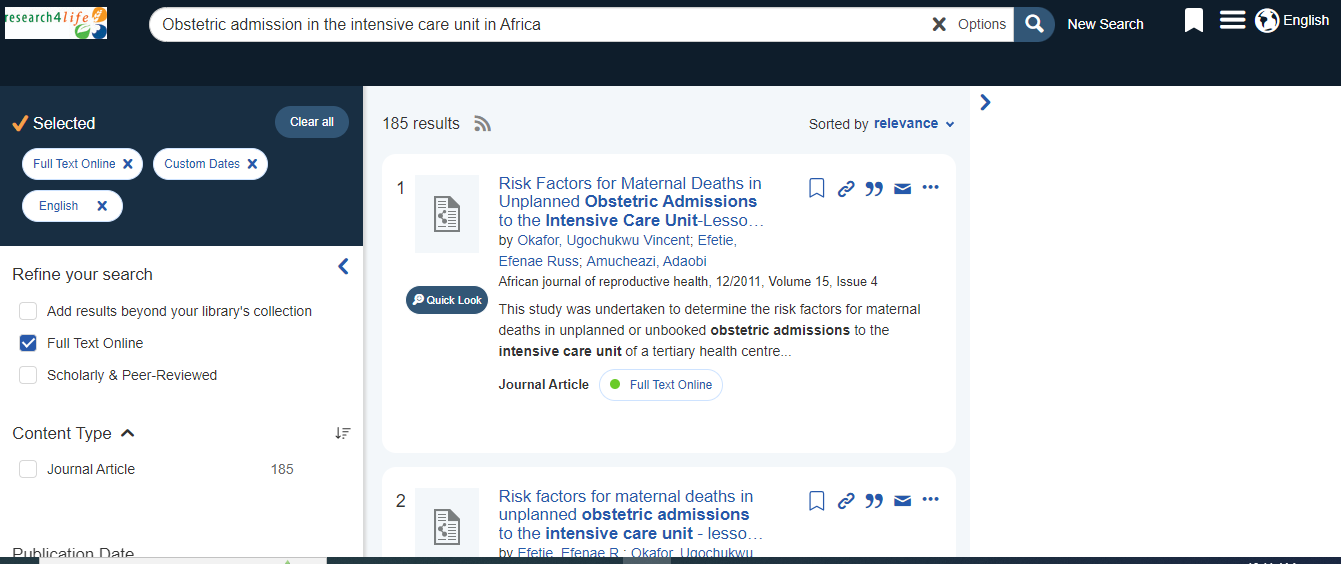

Supplement: S3 File — (DOCX) [file pone.0320254.s003.docx]
