# Supplementary material for: Obstetric admission and maternal mortality in the intensive care unit in Africa: A systematic review and meta-analysis
Source: PLoS One. 2025 Apr 16;20(4):e0320254. doi: 10.1371/journal.pone.0320254 (PMC12002433; doi:10.1371/journal.pone.0320254)
Supplement: S5 File — (DOCX) [file pone.0320254.s005.docx]

| **Table: Showing the list of studies included and excluded from the analysis (46)** | | | | | | |
| --- | --- | --- | --- | --- | --- | --- |
| **S.N** | **Study** | **Year** | **Country** | **Title** | **Eligibility** | **Reason for exclusion** |
| 1 | Study 1 (1) | 2015 | S/Africa | Obstetric intensive care admissions at a tertiary hospital in Limpopo Province, South Africa. | included |  |
| 2 | Study 2 (2) | 2015 | India | Obstetric admissions to the intensive care unit: a five year review | Exclude | Outside side Africa |
| 3 | Study 3 (3) | 2013 | Kenya | Predictors Of Admissions Among Obstetric Patients At The Critical Care Unit, Kenyatta National Hospital | Excluded | Out of our interest |
| 4 | Study 4 (4) | 2018 | Nigeria | Obstetric Admission into the Intensive Care Unit (ICU) of the University of Port Harcourt Teaching Hospital: A Ten-Year Review | Excluded | The study used data from 2006 |
| 5 | Study 5 (5) | 2018 | Nigeria | Pattern and Outcome of Obstetric Admissions into the Intensive Care Unit of a Southeast Nigerian Hospital. | Excluded | Methodological difference |
| 6 | Study 6 (6) | 2018 | India | Study of Obstetric Patients Admitted To Intensive Care Unit (ICU) In a High Volume Tertiary Care Center | Excluded | Outside side Africa |
| 7 | Study 7 (7) | 2011 | Kenya | Analysis of obstetric admission patterns and management in the critical care unit, Kenyatta national hospital | Excluded | Out of our interest |
| 8 | Study 8 (8) | 2011 | Nigeria | Risk Factors for Maternal Deaths in Unplanned Obstetric Admissions to the Intensive Care Unit-Lessons for Sub-Saharan Africa | Excluded | Out of our interest |
| 9 | Study 9 (9) | 2022 | Kenya | Indications, Clinical Characteristics, Management and Outcome of Patients Admitted to a Newly Dedicated Obstetrics Intensive Care Unit at the Kenyatta National Hospital: Descreptive Retrospective Cohort Study | Excluded | Methodological difference |
| 10 | Study 10 (10) | 2017 | Iran | Evaluation of Admission Indications, Clinical Characteristics and Outcomes of Obstetric Patients Admitted to the Intensive Care Unit of a Teaching Hospital Center: A Five-Year Retrospective Review. | Excluded | Outside side Africa |
| 11 | Study 11 (11) | 2022 | Nigeria | Obstetric Patients Requiring Intensive Care: Prevalence, Clinical Characteristics and Outcome in a Tertiary Care Institute in Nigeria. | Included |  |
| 12 | Study 12 (12) | 2019 | S/Africa | The impact of an obstetrician-led, labor ward critical care unit: A prospective comparison of outcomes before and after establishment | Excluded | Out of our interest |
| 13 | Study 13 (13) | 2021 | Rwanda | High mortality rate of obstetric critically ill women in Rwanda and its predictability | Included |  |
| 14 | Study 14 (14) | 2020 | Nigeria | A 5-year review of obstetrics and gynaecology admission into the intensive care unit of a tertiary hospital in Northern Nigeria | Exclude | Methodological difference |
| 15 | Study 15 (15) | 2013 | Nepal | Study of obstetric patients admitted to Intensive Care Unit (ICU) at Kathmandu Medical College Teaching Hospital | Excluded | Outside side Africa |
| 16 | Study 16 (16) | 2016 | Nigeria | Obstetric admissions in a general intensive care unit in north-central Nigeria | Included |  |
| 17 | Study 17 (17) | 2019 | India | Contributory Factors for Obstetric ICU Admission: A Prospective Cross-sectional Study | Excluded | Outside side Africa |
| 18 | Study 18 (18) | 2018 | Korea | Indications and characteristics of obstetric patients admitted to the intensive care unit: a 22-year review in a tertiary care center | Excluded | Outside side Africa |
| 19 | Study 19 (19) | 2018 | India | A study to know clinical characteristics and outcome of obstetrics patients requiring ICU admission | Excluded | Outside side Africa |
| 20 | Study 20 (20) | 2021 | Egypt | Maternal Morbidities and Mortalities: Rates and Indicators at Intensive Care Unit in Woman’s Health Hospital | Excluded | Methodological difference |
| 21 | Study 21 (21) | 2015 | Nigeria | Intensive care management and outcome of women with hypertensive diseases of pregnancy | Included |  |
| 22 | Study 22 (22) | 2022 | Ethiopia | Outcomes and Associated Factors of Mothers Admitted to Intensive Care Unit During Pregnancy and Postpartum at Saint Paul’s Hospital Millennium Medical College  <https://repo.spirhr.org/xmlui/handle/123456789/77> | Included |  |
| 23 | Study 23 (23) | 2019 | Malawi | Obstetric admissions and outcomes in an intensive care unit in Malawi | Included |  |
| 24 | Study 24 (24) | 2021 | Ghana | A ten-year review of indications and outcomes of obstetric admissions to an intensive care unit in a low-resource country | Included |  |
| 25 | Study 25 (25) | 2016 | Nigeria | Severe maternal morbidity in a general intensive care unit in Nigeria: clinical profiles and outcomes | Included |  |
| 26 | Study 26 (26) | 2015 | Cameron | Trend in admissions, clinical features and outcome of preeclampsia and eclampsia as seen from the intensive care unit of the Douala General Hospital, Cameroon | Excluded | Differences in study participant |
| 27 | Study 27 (27) | 2015 | Sudan | Obstetric and gynecologic admissions to the intensive care unit at Khartoum Hospital, Sudan | Excluded | Full text not available |
| 28 | Study 28 (28) | 2015 | Nigeria | Predictors of maternal mortality among critically ill obstetric patients | Excluded | Methodological difference |
| 29 | Study 29 (29) | 2018 | Zimbabwe | Incidence of maternal near miss in the public health sector of Harare, Zimbabwe: a prospective descriptive study | Excluded | Methodological difference |
| 30 | Study 30 (30) | 2022 | Nigeria | A Five-year Review of the Pattern and Outcome of Obstetric Admissions into the Intensive care unit | Included |  |
| 31 | Study 31 (31) | 2017 | S/Africa | Obstetric patients admitted to the intensive care unit of Dr George Mukhari Academic Hospital | Included |  |
| 32 | Study 32 (32) | 2020 | S/Africa | Characteristics and outcomes of obstetric patients with maternal sepsis requiring admission to a South African intensive care unit: A retrospective review | Excluded | Methodological difference |
| 33 |  |  |  |  |  |  |
|  | Study 33 (33) | 2019 | Nigeria | Trends of critical care management of obstetric patients in a tertiary hospital in sub-Saharan Africa | Excluded | Differences in study participants |
| 34 | Study 34 (34) | 2016 | S/Africa | Avoidable factors associated with pregnant and postpartum patients admitted to two intensive care units in South Africa : research | Excluded | Methodological difference |
| 35 | Study 35 (35) | 2022 | Ethiopia | Obstetrics mortality and associated factors in intensive care unit of Addis Ababa public hospital in, 2020/21: A hospital based case control study | Excluded | Methodological difference |
| 36 | Study 36 (36) | 2020 | Ethiopia | Obstetric ICU admissions and their outcomes in Ayder Comprehensive Specialized Hospital: Institution based retrospective study | Excluded | Methodological difference |
| 37 | Study 37 (37) | 2021 | Ethiopia | Factors associated with obstetrics mortality in intensive care unit of Addis Ababa public hospital in, 2020/2021 | Excluded | Duplication |
| 38 | Study 38 (38) | 2011 | Egypt | Clinical characteristics and outcomes of obstetric patients requiring ICU admission. Crit Care | Excluded | Full text not available |
| 39 | Study 39 (39) | 2017 | Egypt | Near-miss cases admitted to a maternal intensive care unit,Alexandria,Egypt | Excluded | Methodological difference |
| 40 | Study 40 (40) | 2022 | Sudan | Admission Criteria of Obstetric Patients in Selected Intensive Care Units, Khartoum State, Sudan | Excluded | Full text not available |
| 41 | Study 41 (41) | 2011 | Kenya | Githae F, Mung’ayi V, Stones W. Course and outcome of obstetric patients admitted to a University Hospital Intensive Care Unit | Excluded | Uses a very small sample size |
| 42 | Study 42 (42) | 2019 | Rwanda | Admission Indications and Outcomes of Obstetric Patients in the Intensive Care Unit (ICU) at the University Teaching Hospital of Kigali (CHUK) | Excluded | Methodological difference |
| 43 | Study 43 (43) | 2019 | Rwanda | Obstetric admissions in Intensive Care Units of University Teaching Hospitals of Butare and Kigali | Excluded | Duplication |
| 44 | Study 44 (44) | 2023 | Somaliland | Facility-based maternal deaths: Their prevalence, causes and underlying circumstances. | Excluded | Methodological difference |
| 45 | Study 45 (45) | 2023 | Somalia | Outcomes of Women with Preeclampsia and Eclampsia Admitted in the Intensive Care Unit at a Tertiary Care Hospital | Excluded | Differences in study participant |
| 46 | Study 46 (46) | 2011 | Morocco | Admissions of women in the third trimester of pregnancy to an intensive care unit in Morocco over a 4-year period | Excluded | Full text is not available |

**References**

1. Ntuli TS, Ogunbanjo G, Nesengani S, Maboya E, Gibango M. Obstetric intensive care admissions at a tertiary hospital in Limpopo Province, South Africa. South Afr J Crit Care. 2015;31(1):8–10.

2. Pattnaik T, Samal S, Behuria S. Obstetric admissions to the intensive care unit: a five year review. Int J Reprod Contracept Obstet Gynecol. 2015;1914–7.

3. Kimani RW. Predictors Of Admissions Among Obstetric Patients At The Critical Care Unit, Kenyatta National Hospital.

4. Green K, Orazulike N. Obstetric Admission into the Intensive Care Unit (ICU) of the University of Port Harcourt Teaching Hospital: A Ten-Year Review. J Adv Med Med Res. 2018 Mar 17;25(9):1–7.

5. Ozumba BC, Ajah LO, Obi VO, Umeh UA, Enebe JT, Obioha KC. Pattern and Outcome of Obstetric Admissions into the Intensive Care Unit of a Southeast Nigerian Hospital. Indian J Crit Care Med Peer-Rev Off Publ Indian Soc Crit Care Med. 2018 Jan;22(1):16–9.

6. Yelamanchili DA, Cherukuri DK. Study of Obstetric Patients Admitted To Intensive Care Unit (ICU) In a High Volume Tertiary Care Center.

7. Kirumwa MA. Analysis of obstetric admission patterns and management in the critical care unit, Kenyatta national hospital.

8. Okafor UV, Efetie ER, Amucheazi A. Risk Factors for Maternal Deaths in Unplanned Obstetric Admissions to the Intensive Care Unit-Lessons for Sub-Saharan Africa. Afr J Reprod Health. 2011;15(4):51–4.

9. Rita A. Indications, Clinical Characteristics, Management and Outcome of Patients Admitted to a Newly Dedicated Obstetrics Intensive Care Unit at the Kenyatta National Hospital: Descreptive Retrospective Cohort Study.

10. Farzi F, Mirmansouri A, Roshan ZA, Nabi BN, Biazar G, Yazdipaz S. Evaluation of Admission Indications, Clinical Characteristics and Outcomes of Obstetric Patients Admitted to the Intensive Care Unit of a Teaching Hospital Center: A Five-Year Retrospective Review. Anesthesiol Pain Med. 2017 May 30;7(3):e13636.

11. Asudo DrFD, Akitoye OA, Abdullahi HI. Obstetric Patients Requiring Intensive Care: Prevalence, Clinical Characteristics and Outcome in a Tertiary Care Institute in Nigeria. EAS J Anaesthesiol Crit Care. 2022 Aug 19;4(4):52–63.

12. Langenegger EJ, Hall D, Mattheyse F, Harvey J. The impact of an obstetrician-led, labor ward critical care unit: A prospective comparison of outcomes before and after establishment. Obstet Med. 2020 Sep 1;13(3):132–6.

13. Rudakemwa A, Cassidy AL, Twagirumugabe T. High mortality rate of obstetric critically ill women in Rwanda and its predictability. BMC Pregnancy Childbirth. 2021 May 25;21(1):401.

14. Takai IU, Ahmed ZD, Umar UA, Galadanci JS, Mohammad AMM. A 5-year review of obstetrics and gynaecology admission into the intensive care unit of a tertiary hospital in Northern Nigeria. P H Med J. 2020 Aug;14(2):51.

15. Saha R, Shakya A. Study of obstetric patients admitted to Intensive Care Unit (ICU) at Kathmandu Medical College Teaching Hospital. J Kathmandu Med Coll. 2013;2(4):196–200.

16. Embu HY, Isamade ES, Nuhu SI, Oyebode TA, Kahansim ML. Obstetric admissions in a general intensive care unit in north-central Nigeria. Trop J Obstet Gynaecol. 2016;33(1):14–20.

17. Srivastava H, Singh S, Srivastava S, Goel N. Contributory Factors for Obstetric ICU Admission: A Prospective Cross-sectional Study. | EBSCOhost [Internet]. Vol. 13. 2019 [cited 2024 Dec 19]. p. 14. Available from: https://openurl.ebsco.com/contentitem/doi:10.7860%2FJCDR%2F2019%2F41609.13102?sid=ebsco:plink:crawler&id=ebsco:doi:10.7860%2FJCDR%2F2019%2F41609.13102

18. Yi HY, Jeong SY, Kim SH, Kim Y, Choi SJ, Oh S young, et al. Indications and characteristics of obstetric patients admitted to the intensive care unit: a 22-year review in a tertiary care center. Obstet Gynecol Sci. 2018 Feb 8;61(2):209–19.

19. Garg P, Tripathi U. A study to know clinical characteristics and outcome of obstetrics patients requiring ICU admission. Int J Reprod Contracept Obstet Gynecol. 2018 Jul 1;7(7):2639–45.

20. Omer Z, Hassan ZE, Mahmoud G, Abdelsalam T. Maternal Morbidities and Mortalities: Rates and Indicators at Intensive Care Unit in Woman’s Health Hospital. Assiut Sci Nurs J. 2021 Dec 1;9(27):10–20.

21. Imarengiaye CO, Isesele TO. Intensive care management and outcome of women with hypertensive diseases of pregnancy. Niger Med J. 2015 Oct;56(5):333.

22. Mideksa T, Mekonnen T, Mengiste B. Outcomes and Associated Factors of Mothers Admitted to Intensive Care Unit During Pregnancy and Postpartum at Saint Paul’s Hospital Millennium Medical College, Addis Ababa, Ethiopia. 2022.

23. Prin M, Kadyaudzu C, Aagaard K, Charles A. Obstetric admissions and outcomes in an intensive care unit in Malawi. Int J Obstet Anesth. 2019 Aug 1;39:99–104.

24. Anane-Fenin B, Agbeno EK, Osarfo J, Anning DAO, Boateng AS, Ken-Amoah S, et al. A ten-year review of indications and outcomes of obstetric admissions to an intensive care unit in a low-resource country. PLOS ONE. 2021 Dec 31;16(12):e0261974.

25. Igbaruma S, Olagbuji B, Aderoba A, Kubeyinje W, Ande B, Imarengiaye C. Severe maternal morbidity in a general intensive care unit in Nigeria: clinical profiles and outcomes. Int J Obstet Anesth. 2016 Dec 1;28:39–44.

26. Priso EB, Njamen TN, Tchente CN, Kana AJ, Landry T, Tchawa UFN, et al. Trend in admissions, clinical features and outcome of preeclampsia and eclampsia as seen from the intensive care unit of the Douala General Hospital, Cameroon. Pan Afr Med J. 2015;21:103.

27. Obstetric and gynecologic admissions to the intensive care unit at Khartoum Hospital, Sudan - Ibrahim - 2015 - International Journal of Gynecology & Obstetrics - Wiley Online Library [Internet]. [cited 2024 Dec 19]. Available from: https://obgyn.onlinelibrary.wiley.com/doi/epdf/10.1016/j.ijgo.2014.10.019

28. Adeniran AS, Bolaji BO, Fawole AA, Oyedepo OO. Predictors of maternal mortality among critically ill obstetric patients. Malawi Med J. 2015 Mar;27(1):16–9.

29. Chikadaya H, Madziyire MG, Munjanja SP. Incidence of maternal near miss in the public health sector of Harare, Zimbabwe: a prospective descriptive study. BMC Pregnancy Childbirth. 2018 Nov 26;18(1):458.

30. John CO, Alegbeleye JO, Oppah IC. A Five-year Review of the Pattern and Outcome of Obstetric Admissions into the Intensive care unit of a University Teaching Hospital in Southern Nigeria. Int J Sci Res Arch. 2022;5(2):155–62.

31. Motiang M. Obstetric patients admitted to the intensive care unit of Dr George Mukhari Academic Hospital, Ga-Rankuwa, South Africa. South Afr J Crit Care. 2017 Aug 2;33(1):12–4.

32. Lafon JY, Buga EC, Nethathe GD. Characteristics and outcomes of obstetric patients with maternal sepsis requiring admission to a South African intensive care unit: A retrospective review. South Afr J Obstet Gynaecol. 2020 Dec;26(3):1–5.

33. Onyekwulu FA, Okeke TC. Trends of critical care management of obstetric patients in a tertiary hospital in sub-Saharan Africa. Int J Res Med Sci. 2019 Apr 26;7(5):1420.

34. Ngene NC, Moodley J, Von RRP, Makinga PN, Paruk F. Avoidable factors associated with pregnant and postpartum patients admitted to two intensive care units in South Africa : research. South Afr J Obstet Gynaecol. 2016 Sep;22(1):8–12.

35. Tasew A, Melese E, Jemal S, Getachew L. Obstetrics mortality and associated factors in intensive care unit of Addis Ababa public hospital in, 2020/21: A hospital based case control study. Ann Med Surg. 2022 Sep 1;81:104458.

36. Teshale Y, Zelelow yibrah B, Adhana MT. Obstetric ICU admissions and their outcomes in Ayder Comprehensive Specialized Hospital: Institution based retrospective study. East Afr J Health Sci. 2020 Jan 1;2(1):250–62.

37. Tasew A. Factors associated with obstetrics mortality in intensive care unit of Addis Ababa public hospital in, 2020/2021. (A hospital based case control study) [Internet]. Addis Abeba University; 2021 [cited 2024 Dec 20]. Available from: http://etd.aau.edu.et/handle/123456789/28112

38. El-Abd H, Mashhour K, Mwafy A. Clinical characteristics and outcomes of obstetric patients requiring ICU admission. Crit Care. 2011;15(Suppl 1):P513.

39. Sultan E, Shehata S, Shaarawy S, Ashry M. Near-miss cases admitted to a maternal intensive care unit,Alexandria,Egypt. East Mediterr Health J. 2017 Oct 1;23(10):694–702.

40. Admission Criteria of Obstetric Patients in Selected Intensive Care Units, Khartoum State, Sudan (2022) – One.Surgery Research Index [Internet]. [cited 2024 Dec 20]. Available from: https://research.one.surgery/admission-criteria-of-obstetric-patients-in-selected-intensive-care-units-khartoum-state-sudan-2022/

41. Githae F, Mung’ayi V, Stones W. Course and outcome of obstetric patients admitted to a University Hospital Intensive Care Unit. East Afr Med J. 2011;88(10):356–60.

42. Tuyishime E, Kalala B, Laaman K, Mershon JP, Uwineza J, Durieux M, et al. Admission Indications and Outcomes of Obstetric Patients in the Intensive Care Unit (ICU) at the University Teaching Hospital of Kigali (CHUK): A retrospective descriptive study. 2019;76.

43. Rudakemwa A. Obstetric admissions in Intensive Care Units of University Teaching Hospitals of Butare and Kigali: Prevalence and Outcomes. Research work submitted in partial fulfillment of the requirements for award of Masters of medicine degree in Anesthesiology [Internet]. 2019 [cited 2024 Dec 20]; Available from: http://dr.ur.ac.rw/handle/123456789/2110

44. Ali Egal J, Essa A, Osman F, Klingberg-Allvin M, Erlandsson K. Facility-based maternal deaths: Their prevalence, causes and underlying circumstances. A mixed method study from the national referral hospital of Somaliland. Sex Reprod Healthc. 2023 Sep 1;37:100862.

45. Hilowle NM, Ahmed SA, Yusuf Ali K, Altinel E, Waberi MM, Hassan MS, et al. Outcomes of Women with Preeclampsia and Eclampsia Admitted in the Intensive Care Unit at a Tertiary Care Hospital in Mogadishu, Somalia. Anesthesiol Res Pract. 2023 Nov 10;2023:6641434.

46. Bentata Y, Housni B, Mimouni A, Abouqal R. Admissions of women in the third trimester of pregnancy to an intensive care unit in Morocco over a 4-year period.
