# Supplementary material for: Obstetric admission and maternal mortality in the intensive care unit in Africa: A systematic review and meta-analysis
Source: PLoS One. 2025 Apr 16;20(4):e0320254. doi: 10.1371/journal.pone.0320254 (PMC12002433; doi:10.1371/journal.pone.0320254)
Supplement: Attachment — Submitted filename: Reviewed Paper.docx [file pone.0320254.s006.docx]

Thanks, Dear Authors for your selecting this interesting topic for the systematic review

**Tittle:** Obstetric Admission admission in the intensive car unit in Africa: a systematic Review and meta-analysis

1) The tittle is good but I would be happy if it included the outcome of the admission that the prognosis of the patient
2) Do you think that sufficient research are there in this area of study?
3) Why you select this topic only in Africa? Do you think that the problem is more in the continent rather than in the country?

**Abstract:**

1) Better to have structured statements like aim, objectives, Results, conclusions and so on
2) How do you suggest about the t existing problem?

**Introduction:**

1) The part of your introduction is too short and what is intention to study it, that is it is obvious that pregnancy has its own complication and why you study the proportion and its indication?
2) The problem is not well stated and it needs major revision and re write clearly before its publication
3) How did you know the quality of care for the pregnant mother by conducting this study?
4) Generally, I am not comfortable by your writing up of the introduction or background that is better to write it by clearly state the magnitude, impact and the factors in this area of problem

**Method:**

1) It is better to said Method and materials than methodology because it is the study of the method rather than explain about the method of the study
2) You preferred that to conduct the systematic review, why you not conduct direct research on this study area or topic?
3) How you intervene	for your finding?

**Result:**

1) The result part seems like simple statement and it lakes clear discussion, please re write as per your topic and findings unless and other wise I doubt to pass for publication

**Conclusion:**

1) Your conclusion was too surface due to that your finding and discussion was poor

Generally there is the question of Nobility of your study finding, your conclusion and recommendation is not satisfactory
