# Supplementary material for: Obstetric admission and maternal mortality in the intensive care unit in Africa: A systematic review and meta-analysis
Source: PLoS One. 2025 Apr 16;20(4):e0320254. doi: 10.1371/journal.pone.0320254 (PMC12002433; doi:10.1371/journal.pone.0320254)
Supplement: Attachment — Submitted filename: Response to Reviewers.docx [file pone.0320254.s009.docx]

**Rebuttal to Editor and Reviewers Comments**

Dear Editor,

We would like to thank you and the reviewers for your insightful comments and suggestions. We have carefully considered your feedback and have made significant revisions to the manuscript.

Editor’s comment:

Title:

**Comment 1**: The manuscript needs to incorporate “the outcomes of obstetric admission in Intensive care unit in Africa in addition to the proportion.”

**Response**: we have incorporated the feedback and revised the title to include the maternal mortality among obstetric ICU patients

Abstract:
**Comment 2**: The background is not well written.

**Response**: we have made revisions to the background section to improve clarity and coherence, ensuring that it effectively sets the context for the study.
**Comment 3**: Knowing the only prevalence of obstetric admission is not important but if you add the quality of intervention with admission is help for the women.

**Response**: we acknowledged that understanding the quality of interventions provided is crucial for improving patient outcomes. Unfortunately, many studies lack detailed information and clarity on the specific interventions implemented and their impact on patient outcomes. For this reason, we couldn’t address this issue but suggested further research to identify evidence-based practices.
**Comment 4**: The method does not include the following points; quality assessment tool and analysis techniques and reviewing protocol were not explained.

**Response**: we have accepted this feedback and addressed the issues by providing a more detailed explanation of the quality assessment tools, analysis techniques, and the reviewing protocol in the methods section.

**Comment 5:** The abstract lacks some of the summary of the study.

**Response:** We have revised the abstract to ensure it provides a comprehensive summary of the study, including key findings and implications.

**Introduction:**

**Comment 6:** The research gaps of the topic are not well explained. Why are you interested in studying the proportion of obstetric admissions?
**Response:** We appreciate this observation. We have revised the introduction to better articulate the research gaps and the significance of studying the proportion of obstetric admissions. This research is crucial for understanding the burden of obstetric conditions in intensive care settings, particularly in Africa, where data is limited.

**Methodology**:
**Comment 7:** The explanation of how articles were retrieved from different databases lacks detail regarding keywords, Medical Subject Headings (MeSH Terms), and Boolean connectors for each specific database.
**Response:** Thank you for this feedback. The search was conducted using the keywords: "Obstetric admission," "Intensive care unit," and "Africa." For each keyword, relevant Medical Subject Headings (MeSH) terms and entry words were utilized. All MeSH terms and entry words were combined using “OR” to retrieve a broad range of studies. Subsequently, the four keywords were combined with each of the elements using “AND” to generate specific and relevant articles. Further details can be found in Supplementary File S3.
**Comment 8: J**ustification for reviewing data from 2009 to 2023?
**Response:** I have clarified this point in the revised manuscript. Our decision to review literature from 2009 to 2023 is based on a previous systematic review that covered the period from 1990 to 2008 in developing regions, including Africa. This timeframe allows us to identify emerging trends and changes in obstetric admissions over the past decade.
**Comment 9:** The details of quality assessment findings were not cited in the manuscript under “Data extraction and quality assessment.”
**Response:** We acknowledge this oversight and have now cited the corresponding supplementary file (S1) in the revised manuscript to provide clarity on the quality assessment findings.

**Result:**

**Comment 10:** Total Number of Retrieved Articles

**Response**: We acknowledge that the total number of articles retrieved from each database was not initially detailed. We have now included this information in the revised manuscript, and you can refer to the PRISMA flow diagram for a comprehensive overview.

**Comment** **11**: Sensitivity Analysis

**Response**: No study was found to significantly influence the pooled estimate

**Comment** 12: Clarity about the justification (lines 185-186)

**Response**: We have made some modifications
**Comment 13**: Implications of the study

**Response**: The implications of our study are as follows:
- Our findings underscore the necessity for improved access to quality maternal healthcare, especially in resource-limited settings.
- The study highlights the critical importance of early identification and management of obstetric complications to prevent severe outcomes and minimize the need for ICU admissions.
**Comment 14**: Use of PRISMA 2020 Flow Diagram

**Response**: We would like to confirm that we have utilized the PRISMA 2020 flow diagram in our manuscript. Please refer to Figure 1 for the relevant details.

Reviewer #1 comment:

**Abstract**

**Comment 1:** Title Revision

**Response**: In response to the suggestion regarding the title, we have revised it to include "maternal mortality among obstetric ICU patients," thereby emphasizing the critical outcomes of our study.
**Comment** 2: Survival Rate Assessment

**Response**: While we acknowledge the importance of assessing survival rates among mothers admitted to the ICU as a measure of care quality, we have opted to focus on maternal mortality rates. This decision is based on the prevalence of studies reporting mortality rates, which provides a more standardized metric for comparison across different settings.
**Comment** 3: Morbidity and Mortality Indicators

**Response**: We have made some modifications that we have clarified in the manuscript how obstetric admissions to the ICU can improve maternal outcomes

**Comment** 4: Correction of Data

**Response**: We have corrected the statement regarding the total number of mothers admitted to the ICU to reflect "a total of 10,190 mothers admitted to the intensive care unit," as per your feedback.
**Comment** 5: Staff Quality and Equipment Gaps

**Response**: We have revised the section accordingly.
**Comment** 6. Keywords Clarification

**Response**: We have revised the keywords to eliminate redundancy, ensuring that terms such as "obstetric," "pregnancy complications," "critical illness," and "severe maternal morbidity" are used appropriately without overlap.

**Introduction**
**Comment** 7. References Addition

**Response**: We have added the necessary references to support the claims made in the specified paragraph, enhancing the manuscript's credibility.
**Comment** 8. Admission Rates in Developed Nations

**Response**: We have included specific figures regarding the lower admission rates of critically ill obstetric patients in developed nations, providing a clearer context for comparison. For instance, we noted that in developed nations, the admission rate ranges from 0.08% to 0.76%.

**Methodology**

**Comment 9.** Inclusion of Unpublished Studies
**Response**: We included unpublished studies in our systematic review to mitigate publication bias and to present a more comprehensive overview of the research topic. By incorporating unpublished data, we ensure that all relevant evidence is considered, which contributes to more accurate conclusions.
**Comment 10.** Rationale for Starting from 2009 and the Inclusion of Studies from 2019 **Response:** Although our systematic review was initially designed to encompass studies from 2009, our search strategy and inclusion criteria ultimately identified relevant studies commencing from 2019. The exclusion of earlier studies was based on specific quality and relevance criteria rather than publication date alone, as many did not meet the rigorous methodological standards we established.
**Comment 11.** Quality Categorization of Included Articles
**Response**: Thank you for your inquiry regarding the quality categorization of the included articles. In our analysis, we focused on articles categorized as having high quality (80% or higher) or moderate quality (65%–80%). Articles rated as low quality (less than 65%) were excluded from the analysis to ensure that our findings are grounded in studies that adhere to acceptable methodological standards.

**Result**

**Comment** 12: Add obstetric Outcomes after Associated Factors for admission.

**Response**: While many studies have focused on the immediate causes of obstetric ICU admission, such as hemorrhage, preeclampsia, and sepsis, it's important to recognize that broader factors can influence both the risk of these complications and patient outcomes. However, many studies lack detailed information on these factors. Therefore, while our review primarily focuses on the immediate causes of admission, we appreciated your suggestion and emphasized the importance of future research that investigates the role of socioeconomic factors, healthcare system factors, and other contextual variables influencing obstetric ICU admission and outcomes.

**Comment 13:** Clarification of Non-Obstetric Causes in Table **4**
**Response**: Thank you for pointing out the need for clarification regarding other non-obstetric causes. We have revised Table 4 to include a parenthetical note listing additional causes such as anesthesia-related complications, pulmonary edema, fatty liver of pregnancy, anemia, coagulopathy, and kidney injury, as referenced in various studies.

**Discussion**
**Comment 14:** Discussion (Lines 180-181): Maternal Survival Rates Post-ICU Admission
**Response**: We appreciate your insight regarding the survival rates of mothers admitted to the ICU for obstetric complications. In response, we have expanded our discussion to include an assessment of maternal mortality rates following ICU admission, thereby addressing this critical aspect of our findings.
**Comment 15:** Lines 188-190: Comparison of Pooled Results with Primary Study Results
**Response**: We acknowledge the importance of comparing our pooled results with national findings or other systematic reviews. However, we must clarify that, to date, there are no systematic reviews or national datasets that directly align with our findings for comparison.
**Comment 16:** Conclusion (Lines 217-219): Gaps in Health Education
**Response**: Your recommendation regarding potential gaps in health education is well taken.
**Comment 17:** Strengths and Limitations of the Study
**Response**: We appreciate your suggestion to include a section on the strengths and limitations of our study. This has been added to the revised manuscript, and many improvements have been made.

Reviewer #2 comments

**Title**

**Comment** 1. Title Revision

**Response**: We acknowledged the suggestion to revise the title to include the outcome and revise it as ‘obstetric admission and maternal mortality among obstetric ICU patients in Africa’.
**Comment** 2. Research Sufficiency

**Response**: Regarding the concern about the sufficiency of research in this area, our literature review revealed a significant number of studies primarily focusing on patient admission.
**Comment** 3. Geographic Focus

**Response**: The decision to focus on Africa stems from the region's disproportionate burden of maternal mortality. While maternal mortality is indeed a global concern, the unique challenges faced in Africa—such as limited access to quality healthcare, inadequate infrastructure, and socio-economic disparities—warrant a concentrated examination of this issue within the continent.
**Abstract**
**Comment 4**: Abstract Structure

**Response**: In response to the suggestion for a more structured abstract, we have revised the abstract to include clearly defined sections: Background, Objectives, Method, Results, and Conclusions.
**Comment 5:** Suggestions to address existing problems

**Response**: To address the existing challenges in maternal health, we recommended enhancing emergency obstetric care services, and expanding access to obstetric ICU services. Additionally, further research on the effectiveness of various interventions in obstetric ICU care is essential.

**Introduction**

**Comment** 6. Introduction Length and Intent

**Response**: We acknowledge your observation. The intention was to provide a concise overview while highlighting the significance of studying pregnancy complications. We expanded this section to better articulate the rationale behind my research.
**Comment 7**. Problem Statement

**Response**: We have appreciated your feedback on the clarity of the problem statement. We undertook a thorough revision to ensure that the problem is articulated more clearly, providing a solid foundation for the study.
**Comment** 8: Writing Quality of Introduction/Background

**Response**: We understand your concerns about the clarity and comprehensiveness of the introduction. We have revised this section to include a more detailed discussion.

**Method**

**Comment** 9. Terminology

**Response**: We acknowledge your suggestion to use "Methods and Materials" instead of "Methodology."
**Comment** 10. Research approach

**Response**: You raised a valid question regarding the preference for conducting a systematic review over direct research in this area. While direct research is essential for generating new insights, there is a substantial body of primary studies available concerning obstetric ICU patients. A systematic review allows us to synthesize this existing evidence, identify patterns, and highlight gaps in the current research landscape. This structured approach not only enhances our understanding of the existing knowledge but also aids in identifying inconsistencies and areas that require further investigation. Ultimately, this can inform evidence-based decision-making and improve patient outcomes in obstetric intensive care.
**Comment** 11: Intervention Strategies

**Response**: In response to your inquiry about how I plan to intervene based on the findings, I propose several strategies:
- Advocate for policies and regulations that reflect the review's findings.
- Identify and emphasize areas requiring further research, particularly those involving understudied populations or specific interventions.
- Acknowledge the limitations of the review and potential biases to ensure transparency.
- Aim to publish the findings in high-impact journals to reach a broad audience of researchers, clinicians, and policymakers.
- Present the findings at relevant conferences to engage with the scientific community and foster discussions with experts.
- Develop new research proposals or protocols to address identified gaps and generate additional evidence.
- Commit to continuously improving the quality of reviews by adhering to rigorous methodological standards and employing advanced statistical techniques.

**Results**

**Comment 12:** Incorporate necessary depth and discussion
**Response**: We acknowledged that the initial version of the results section lacked the necessary depth and discussion. In response, we have rewritten this section to provide a detailed interpretations and contextualized the results within the broader scope of the field, ensuring that the implications of the findings are clearly articulated.
**Comment 13: Conclusion Section**
**Response**: We understand the concerns raised about the surface-level nature of the conclusion. We have revised this section to better reflect the significance of the study's findings. The updated conclusion now includes a thorough discussion of the implications of the results, addressing the nobility of the study and providing actionable recommendations based on the findings.

Thank you for your valuable feedback, which has significantly contributed to the improvement of this manuscript. We look forward to your response and hope for a favorable consideration of the revised submission.

Sincerely,

Alemwork Abie (corresponding author)
