# Supplementary material for: Obstetric admission and maternal mortality in the intensive care unit in Africa: A systematic review and meta-analysis
Source: PLoS One. 2025 Apr 16;20(4):e0320254. doi: 10.1371/journal.pone.0320254 (PMC12002433; doi:10.1371/journal.pone.0320254)
Supplement: Attachment — Submitted filename: PONE-D-24-22852R1-OA.docx [file pone.0320254.s008.docx]

**Review manuscript PONE-D-24-22852R1, entitled "Obstetric admission and maternal mortality in the intensive care unit in Africa: a systematic review and meta-analysis**

**Key Comments & Suggested Revisions**

**1. Abstract**

- **Strengths:**
- Clearly presents the study’s objectives, methodology, and key findings.
- The statistical results are well-articulated.

**Suggestions:**

- Improve grammar and readability.
- Summarize key qualitative findings.
- Provide clearer interpretation of statistical results.

**2. Introduction**

**Suggestions:**

- Add country-specific maternal mortality and ICU admission rates.
- Explicitly state the research gap and justification.
- Strengthen policy implications.

**3. Methods**

**Suggestions:**

- Justify the mixed-methods approach.
- Provide more details on qualitative data analysis.
- Explain statistical assumptions and ethical considerations.

**4. Results**

**Suggestions:**

- Integrate qualitative and quantitative findings for a more coherent discussion.
- Report all confidence intervals and p-values consistently.
- Interpret effect sizes meaningfully.

**5. Discussion**

**Suggestions:**

- Improve integration of qualitative and quantitative findings.
- Expand the limitations section.
- Strengthen the discussion on policy implications.

**6. Conclusion**

**Suggestions:**

- Strengthen policy connections.
- Acknowledge study limitations in the conclusion.

**7. Language & Formatting Issues**

**Suggestions:**

- Professional proofreading & language editing is strongly recommended.
- Improve paragraph structure for better readability.

**Key Actions Before Resubmission:**

1. Improve language clarity & proofread thoroughly.
2. Strengthen integration of qualitative and quantitative findings.
3. Clarify statistical methodology and reporting.
4. Expand ethical considerations, limitations, and policy implications.
5. Improve the depth and coherence of the discussion section.
